# The role of microRNAs in the pathogenesis, grading and treatment of hepatic fibrosis in schistosomiasis

**DOI:** 10.1186/s13071-019-3866-0

**Published:** 2019-12-30

**Authors:** Qianglin Chen, Jianqiang Zhang, Ting Zheng, Hui Chen, Hao Nie, Bing Zheng, Quan Gong

**Affiliations:** 1 Department of Immunology, School of Medicine, Yangtze University, Jingzhou, Hubei Province 434023 People’s Republic of China; 2 Clinical Molecular Immunology Center, School of Medicine, Yangtze University, Jingzhou, Hubei Province 434023 People’s Republic of China

**Keywords:** Schistosomiasis, MicroRNA, Hepatic fibrosis, Hepatic stellate cells, Biomarker

## Abstract

Schistosomiasis is a prevalent parasitic disease worldwide. The main pathological changes of hepatosplenic schistosomiasis are hepatic granuloma and fibrosis due to worm eggs. Portal hypertension and ascites induced by hepatic fibrosis are usually the main causes of death in patients with chronic hepatosplenic schistosomiasis. Currently, no effective vaccine exists for preventing schistosome infections. For quite a long time, praziquantel (PZQ) was widely used for the treatment of schistosomiasis and has shown benefit in treating liver fibrosis. However, drug resistance and chemical toxicity from PZQ are being increasingly reported in recent years; therefore, new and effective strategies for treating schistosomiasis-induced hepatic fibrosis are urgently needed. MicroRNA (miRNA), a non-coding RNA, has been proved to be associated with the development of many human diseases, including schistosomiasis. In this review, we present a balanced and comprehensive view of the role of miRNAs in the pathogenesis, grading, and treatment of schistosomiasis-associated hepatic fibrosis. The multiple regulatory roles of miRNAs, such as promoting or inhibiting the development of liver pathology in murine schistosomiasis are also discussed in depth. Additionally, miRNAs may serve as candidate biomarkers for diagnosing liver pathology of schistosomiasis and as novel therapeutic targets for treating schistosomiasis-associated hepatic fibrosis.
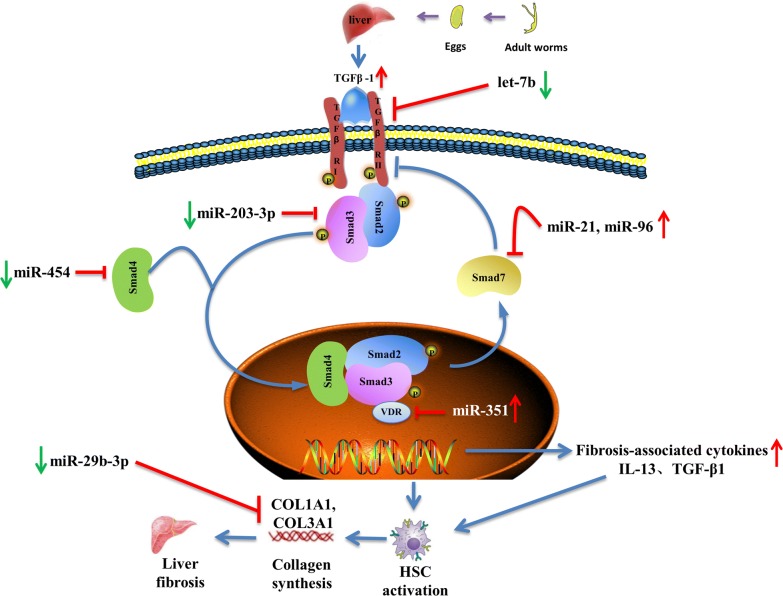

## Background

Schistosomiasis is one of the most common zoonotic parasitic diseases worldwide [1, 2]. It is estimated that at least 230 million people are infected with schistosomes globally [3]. The main species of schistosome that infect humans include *Schistosoma japonicum*, *Schistosoma mansoni* and *Schistosoma haematobium* [4]. In China, *S. japonicum* infection is the main cause of schistosomiasis; this species also presents more severe pathogenicity because it produces more eggs than other *Schistosoma* species [2, 5]. *Schistosoma japonicum* and *S. mansoni* colonize the mesenteric veins, where their eggs induce a local liver granulomatous response and subsequent progression of hepatic fibrosis; this review will focus on *S. japonicum* and *S. mansoni*-induced schistosomiasis. Generally, the development of schistosomiasis can be divided into the acute and chronic stages [6]; the acute phase is believed to be associated with Th1 response [7]. Then a rapid transition from Th1 response into Th2-dominated response will occur when a large number of schistosome eggs are deposited [8]. The main pathological damages incurred during schistosomiasis are granuloma formation and subsequent hepatic fibrosis induced by the eggs in the middle and late stages of infection, which result from the hostʼs immune response to the soluble egg antigen (SEA) [9].

The liver is composed of hepatic parenchymal and non-parenchymal cells. Non-parenchymal cells include hepatic stellate cells (HSCs), Kupffer cells, and liver sinusoidal endothelial cells (LSECs) [10]. Activated HSCs are thought to be central effector cells during hepatic fibrosis [11]. During schistosomiasis, the inflammatory granulomas initially form around the schistosome eggs, then the dormant HSCs are activated by various cytokines and transform into myofibroblast cells to initiate hepatic fibrosis [5, 12]. Hepatic fibrosis, characterized by excessive deposition of extracellular matrix (ECM) [13], is a wound healing response to multiple pathogenic factors such as parasitic infection, alcohol, viruses, cholestasis and oxidative stress [14]. Hepatic fibrosis can further develop into portal hypertension and ascites, which are usually the leading causes of death in patients with schistosomiasis [15]. Praziquantel (PZQ) is the primary drug for treating schistosomiasis. Although PZQ has been shown to aid in ameliorating liver fibrosis *via* multiple mechanisms in murine models of schistosomiasis [16, 17], PZQ cannot completely reverse the progression of chronic liver fibrosis, and the excessive reliance on this drug to treat schistosomiasis has raised concerns about drug resistance [18]. Therefore, effective therapeutic methods for treating schistosomiasis-associated hepatic fibrosis are urgently needed [19].

MicroRNAs (miRNAs), a class of short and non-coding RNAs, have a strong regulatory effect on posttranscriptional gene expression [20]. miRNAs can bind to the 3’-untranslated region (UTR), coding sequence, and 5’-UTR of the target gene mRNA, and mediate mRNA degradation or inhibit its translation [4, 21, 22]. miRNAs are thought to be involved in numerous biological processes such as cell growth, development, proliferation, differentiation, and body metabolism [23, 24]. Numerous studies have shown that miRNA deregulation is related to many human diseases, such as cancers, autoimmune diseases, and parasitic diseases [25–28]. Several studies have also revealed that host miRNAs are differentially expressed before and after *S. japonicum* infection in mouse models, and the expression levels are upregulated or downregulated [29]. Further studies have shown that these differentially expressed miRNAs play modulatory roles in maintaining equilibrium in immune responses, including hepatic granuloma formation and schistosomiasis-induced fibrosis [30]. Here, we focus on recent studies evaluating the roles of miRNAs in the pathogenesis of schistosomiasis-associated liver fibrosis (Table 1). We also discuss the use of miRNAs as diagnostic biomarkers for schistosomiasis-associated hepatopathology progression and the potential of miRNAs as novel therapeutic targets for treating schistosomiasis-associated hepatic fibrosis.

**Table 1:** The role and underlying regulatory mechanisms of miRNAs in the pathogenesis of hepatic fibrosis in schistosomiasis

Function	Type	Target	Mechanism/pathway	References
Pro-fibrosis	miR-21, miR-96	Smad7	SMAD signaling pathway	[40, 41]
	miR-351	VDR	SMAD and IFN-γ signaling pathway	[45]
	miR-146a/b	STAT1	Regulates the transformation of macrophages from M1 to M2/IFN-γ signaling pathway	[46]
	miR-27b	PPARγ	Enhances the activation of hepatic stellate cells	[89]
Anti-fibrosis	miR-203-3p	IL-33/Smad3	IL-33/IL-13 pathway	[67, 70]
	let-7b	TβRI	TGF-β**/**SMAD signaling pathway	[80]
	miR-182	unknown	Preserves Tregs stability and suppressor function	[88]
	miR-15b, miR-16	Bcl2	Caspase signaling pathway	[90]
	miR-454	Smad4	SMAD signaling pathway	[91]
	miR-155	FOXO3a	ERK1 signaling pathway, EMT process	[92, 93]
	miR-29b-3p	COL1A1, COL3A1	TGF-β signaling pathway	[94]
	miR-92a-2-5p	TLR2	Promotes NIH-3T3 cell apoptosis	[95]

## Pro-fibrogenic role of miRNAs in schistosomiasis

### MiR-21 and miR-96 activate the SMAD signaling pathway to promote schistosomiasis-associated hepatic fibrosis

It is well known that severe hepatic fibrosis results when multiple signaling pathways trigger HSC activation [31], and miRNAs can balance multiple growth factor receptor signals during HSC activation [32]. It has been reported that miR-21 is overexpressed in many liver diseases and is considered to be one of the most significantly upregulated miRNAs in activated HSCs in several fibrosis disease models [33]. For example, miR-21 can mediate LX-2 cell activation during liver fibrosis *via* the PTEN/Akt pathway [34]. Also miR-21 has been associated with cholestatic liver injury and liver necrosis disease by activating HSCs and promoting hepatic fibrosis in a murine model of bile duct ligation [35]. It has also been reported that miR-96 can facilitate cell proliferation, migration and invasion by targeting SOX6 in hepatocellular carcinoma [36, 37].

Cumulative evidence suggests that miR-21 and miR-96 are involved in regulating schistosomiasis-associated liver fibrosis partially through the transforming growth factor beta 1 (TGF-β1)/SMAD signaling pathway, which is considered to be the classical signaling pathway during schistosomiasis-associated liver fibrosis [38]. Previous studies found that in murine schistosomiasis, the levels of two major hepatic fibrosis mediators, interleukin (IL)-13 and TGF-β1, were elevated and capable of driving the HSC activation [39]. TGF-β1 promotes miR-21 expression in HSCs by activating SMAD2 and 3 proteins, whereas IL-13 facilitates miR-21 expression by activating the SMAD 1/5, 2, and 3 proteins [40]. Unlike miR-21, increased miR-96 expression in HSCs is primarily mediated by TGF-β1. Specifically, TGF-β1 elevates miR-96 levels by activating SMAD proteins and inducing formation of a complex composed of SMAD2/3, pri-miR-96, and the subunit of the microprocessor complex, DROSHA. SMAD7, a SMAD-signaling regulator, is a common target of miR-21 and miR-96 in schistosomiasis-associated hepatic fibrosis. Surprisingly, miR-21 and miR-96 together exert a synergistic inhibitory effect on SMAD7. Collectively, miR-21 and miR-96 could promote schistosomiasis-associated liver fibrosis by targeting SMAD7 to activate the SMAD signaling pathway and increase collagen expression [40, 41].

### MiR-351 promotes hepatic fibrosis by targeting the vitamin D receptor (VDR) in schistosomiasis

MiR-351 has been shown to participate in modulating various physiopathological processes, including the development of the nervous system and skeletal muscle atrophy, proliferation, and differentiation [42, 43]. For example, upregulated miR-351 improves skeletal muscle atrophy and plays a protective role during acute sepsis by blocking the Tead-4-mediated Hippo signaling pathway [44]. Moreover, miR-351 has been reported to be associated with several liver diseases as an antiviral miRNA [43]. A recent study found that miR-351 mediated schistosome-induced hepatic fibrosis by targeting VDR [45]. Interferon (IFN)-γ negatively regulates miR-351 in HSCs in the early stages of schistosomiasis. IFN-γ inhibits miR-351 production to increase the expressions of VDR and SMAD7, two TGF-β/SMAD signaling channel antagonists, thereby blocking activation of HSCs [45]. The inhibitory role of IFN-γ mainly depends on the signal transducer and activator of transcription 1 (STAT1) and IFN-regulatory factor 2 (IRF2), which are important transcription factors in the IFN-γ signaling pathway. When eggs are deposited in the liver, the secreted cytokines switch from the Th1-type to the Th2-type, and correspondingly, the IFN-γ levels decrease. This weakens the negative regulation of miR-351; thus, the increased miR-351 induces HSC activation and promotes Col1α1, Col3α1, and α-SMA production by targeting VDR [45].

### MiR-146a/b plays a protective role in hepatic schistosomiasis by regulating differentiation of macrophages into M2 cells

M1 macrophages are mainly involved in inflammation and tissue damage by producing pro-inflammatory cytokines such as tumor necrosis factor alpha (TNF-α), IL-1β, IL-12 and IL-23 [46, 47], whereas, M2 macrophages are thought to be important regulatory factors in attenuating excessive inflammation and promoting protective responses of the host mainly by secreting cytokines such as TGF-β and IL-10 [48, 49]. IL-10 is thought to play an immunosuppressive role in infectious diseases and antagonize M1 macrophage-induced tissue damage [50, 51]. Arg-1, the canonical marker of M2 macrophages, is supposed to exhibit both anti-inflammatory and anti-fibrotic activity after infection with *S. mansoni*, and Arg1-expressing macrophages act as critical mediators to downmodulate the immune response in chronic schistosomiasis [52]. Interestingly, M2 macrophages can also expedite fiber dissolution by secreting matrix metallopeptidase (MMPs) [53].

It has been confirmed that macrophages play vital roles in the pathogenesis of schistosomiasis [54]. In the early stages of schistosomiasis, larval and adult worm migration in the host induces a Th1 response, which is characterized by elevated levels of IFN-γ [55], which can induce differentiation of M1 macrophages [56]. After 4–6 weeks of infection, the worm eggs are released, causing a rapid transition from a Th1 to Th2 response in the host [57], and the series of Th2-type cytokines induce miR-146b expression by activating STAT3/6 in macrophages. While miR-146b inhibits the differentiation of macrophage into M1 by targeting STAT1, which is a key component of the IFN-γ signaling pathway [46]. Thus, miR146a/b plays a protective role against hepatic schistosomiasis by regulating macrophage differentiation from M1 to M2 cells [46, 58]. In addition to its role in schistosomiasis, miR146a/b regulates the transformation from liver fibrosis to cirrhosis in patients infected with hepatitis B [59] and attenuates liver fibrosis in carbon tetrachloride (CCL4)-induced rats [60]. It has also been reported that miR-146a/b can regulate macrophage activation by acting on the toll-like receptor family, GM-CSF, M-CSF, and other signaling pathways and molecules [61]. Whether miR-146a/b can also activate macrophages, *via* these mechanisms in schistosomiasis remains unclear.

## Anti-fibrogenic role of miRNAs in schistosomiasis

### Role of miR-203-3p in inhibiting schistosomiasis-induced liver fibrosis

The IL-33/IL-13 pathway is associated with the immunopathological process of liver fibrosis, and hepatic group 2 innate lymphoid cells (ILC2s) have been identified as fibrogenic immune cells in the murine liver [62]. It has been reported that IL-33 promotes fibrosis in many organs, including the liver [63], lungs [64], kidney [65] and heart [66]. In a murine model of schistosomiasis, schistosome eggs trapped tissue induced downregulation of miR-203-3p in HSCs. Correspondingly, IL-33, the target of miR-203-3p, was increased [67]. As an inducer of type 2 immunopathology, IL-33 and its receptor ST2 were shown to be involved in fibrosis development after *S. japonicum* infection by triggering the release of IL-5 and IL-13 [68]. IL-33 promotes proliferation of ILC2s, which secrete large amounts of IL-13, and then IL-13 activates HSCs to produce excessive ECM through the STAT6 pathway [67]. Meanwhile, IL-13 can activate macrophages into M2-types, which promote synthesis of liver collagen and subsequent hepatic fibrosis [69]. Therefore, miR-203-3p can inhibit the process of schistosomiasis-associated liver fibrosis by inhibiting IL-33 secretion. Additionally, an *in vitro* study demonstrated that miR-203 may inhibit the synthesis and deposition of ECM components to prevent HSC activation by targeting SMAD3 [70] and functions to inhibit myocardial fibrosis [71].

### Let-7b inhibits liver fibrosis in schistosomiasis through multiple mechanisms

Let-7 miRNA was originally discovered in the free-living nematode *Caenorhabditis elegans* [72]. Growing evidence suggests that let-7b, one of twelve members of the let-7 family, is associated with a variety of diseases, including tumor, liver, and skin diseases [73]. Let-7b regulates tumorigenesis and cancer progression by inhibiting cell proliferation [74] in thyroid cancer [75], breast cancer [76] and acute lymphoblastic leukemia [77]. Furthermore, let-7b is believed to upregulate the gene expression of heme oxygenase-1 through targeting Bach1 and thus alleviate oxidative damage of human hepatocytes [78]. Let-7b also inhibits the progression of alcoholic liver fibrosis by targeting LIN28B and HMGA2 [79]. Recent studies have shown that let-7b inhibits schistosomiasis-associated liver fibrosis by targeting TβRI, which is considered an important target for inhibiting liver fibrosis [80]. Moreover, let-7b can simultaneously suppress liver fibrosis by inhibiting Th1 and Th2 responses as well as expression of TGF-β1, α-SMA and collagen I [80].

### MiR-182 may regulate the specialization of regulatory T cells in schistosome infections

Regulatory T cells (Tregs), a subgroup of T cells, can maintain immunological tolerance to self-antigens, thereby preventing autoimmune diseases [81]. Tregs exert immunosuppressive effects by secreting inhibitory cytokines, such as TGF-β, IL-10 and IL-35 [82]. Although multiple regulatory cell types have been identified, Tregs remain the most important immunoregulatory cell population to efficiently limit schistosome-induced immunopathological damage to host organs [83, 84]. In schistosomiasis, Tregs can exert their immunosuppressive effects by producing IL-10 to inhibit Th1 response and limit the excessive effects of Th2 response [85]. Th2 and Th17 cells are also reported to upregulate granuloma formation by secreting IL-4 and IL-17, respectively; however, Tregs downregulate the formation of granuloma [86]. Previous studies have shown that miRNAs can affect Tregs generation and plasticity, thereby regulating the pathogenesis and treatment of autoimmune diseases and cancers [87]. A recent study found that miR-182 plays a similar regulatory role during schistosome infections [88]. Local environmental factors, such as IL-4, regulate the miR-182 pathway, thus shaping Th2 into Tregs and preserving Tregs stability and suppressor functions. Although miR-182 is an important mediator of Tregs specialization and stability during schistosome infections, the role and underlying mechanism of miR-182 in the immunopathology of schistosomiasis remain unknown [88].

### Other miRNAs associated with liver fibrosis in schistosomiasis

**Fig. 1:**
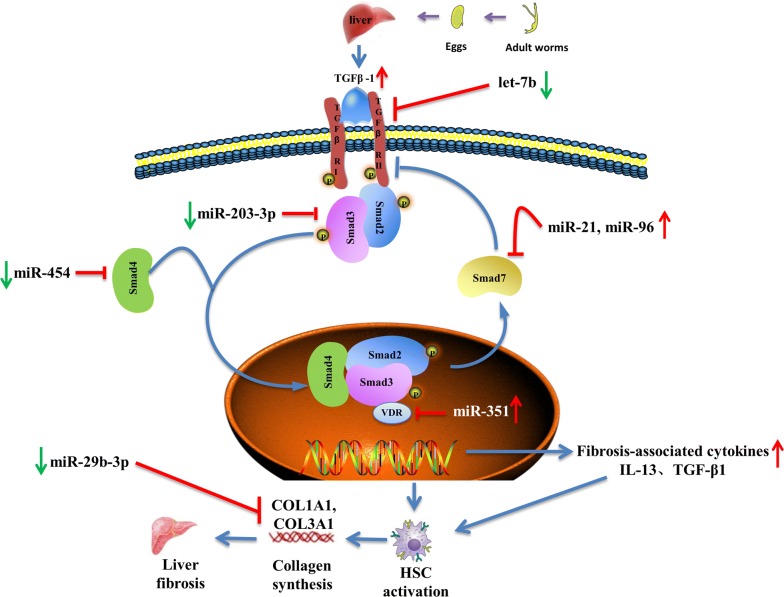
miRNAs regulate the HSC activation and schistosomiasis liver fibrosis through modulating TGF-β/SMAD signaling pathway. The key event of schistosomiasis liver fibrosis is the HSC activation, where the TGF-β/SMAD signaling pathway plays a vital role in the process. After schistosome infection, soluble egg antigen (SEA) induces macrophages to secret TGF-β [119, 120], which is the classic fibrogenic cytokine that promotes the activation of HSC. TGF-β binds to the receptors leading to phosphorylation of Smad-2 and Smad-3, followed by aggregation with Smad-4 and subsequently drives the expression of Smad-7 which negatively regulates TGF-β/SMAD signaling by blocking the TGF-β type I receptor (TβRΙ). Upon HSC activation, synthesis of ECM proteins is enhanced, especially collagen I and II, therefore resulting in liver fibrosis. Dysregulation of miRNAs regulate the TGF-β/SMAD signaling pathway to influence the activation of HSC and therefore exert a pro-fibrosis (miR-21, miR-96 and miR-351) or anti-fibrosis (miR-203-3p, miR-454, let-7b and miR-29b-3p) role in schistosomiasis

In addition to the aforementioned miRNAs, some other miRNAs are also involved in the development of liver fibrosis by regulating HSC activation and apoptosis. For example, an *in vitro* study showed that miR-27b expression is downregulated in rSjP40-treated LX-2 cells, and miR-27b could promote HSCs activation through targeting PPARγ, which is thought to inhibit fibrosis by holding HSCs in a more quiescent phenotype [89]. However, the pro-fibrogenic effect of miR-27b in murine schistosomiasis remains unconfirmed. Also, miR-15b and miR-16 play important roles in inducing HSC apoptosis by targeting bcl-2 in the caspase signaling pathway [90]; miR-454 was reported to be downregulated in *S. japonicum*-induced liver fibrosis models and it could participate in inhibiting HSC activation during schistosomiasis-associated liver fibrosis by targeting Smad4 [91]. As a pleiotropic modulator, miR-155 inhibits HSC activation by blocking the ERK1 signaling pathway [92] and inhibits LX-2 cell activation by targeting FOXO3a [93]. During schistosomiasis-associated liver fibrosis, miR-29b-3p is believed to inhibit HSC activation by targeting COL1A1 and COL3A1 in the TGF-β1 signaling pathway [94]. Moreover, another recent study showed that mmu-miR-92a-2-5p inhibits schistosome-induced liver fibrosis *in vitro* and *in vivo* by targeting TLR2, but the underlying molecular mechanism remains unclear [95]. TGF-β/SMAD signaling is an important pathway for HSC activation, and the modulatory roles of miRNAs in this signaling pathway further affect schistosomiasis-associated liver fibrosis are summarized in Fig. 1.

## Using miRNAs to grade schistosomiasis-associated hepatic fibrosis

It is known to us that the current challenge in treating schistosomiasis is determining how to completely prevent liver fibrosis progression and other immunopathological damage caused by the parasite eggs in the later stage of schistosomiasis [96]. Therefore, effective methods for early diagnosis and grading hepatic fibrosis must be developed. Circulating and exosomal miRNAs could be used as markers for diagnosis or indicators for determining severity of certain diseases and therapeutic effects [97–99]. For example, elevated miR-21, miR-122 and miR-223 in the serum may serve as new biomarkers for liver injury diseases such as liver cancer and chronic hepatitis [100]. MiR-122 and miR-192 were elevated in the serum of mice with drug-induced liver injury [101].

Evidence indicates that host miRNAs in schistosomiasis serve as important molecules for host-parasite interactions and may serve as new biomarkers for diagnosing schistosomiasis and assessing the severity of liver pathology [102]. MiR-223 is reported to be significantly upregulated in the serum of *S. japonicum*-infected mice and is highly associated with hepatic pathological changes. After PZQ treatment in these mice, the miR-223 levels returned to nearly normal, suggesting that miR-223 could be used as a diagnostic biomarker of schistosome infection and a prognostic marker to monitor therapeutic effects [103]. Serological differences in the circulating host miRNAs (miR-122, miR-21 and miR-34a) were tested in a mouse model before and after schistosome infection [104]. Although their respective potential value as biomarkers for diagnosing schistosomiasis was limited, a combination of several biomarkers could be used to evaluate hepatopathology progression in murine schistosomiasis [104]. A subsequent study was performed to test the correlation between the levels of circulating miRNAs and fibrosis grading in human schistosomiasis [105]. Cai et al. [105] evaluated the potential of ten miRNAs in distinguishing the severity of liver fibrosis in *S. japonicum*-infected mice and in schistosomiasis patients and found that four circulating miRNAs (miR-150-5p, let-7a-5p, let-7d-5p and miR-146a-5p) had moderate diagnostic value to discriminate mild from severe liver fibrosis in schistosomiasis patients. Additionally, miR-150-5p displayed the best diagnostic performance for grading hepatic fibrosis [105]. Although miR-706 and miR-134-5p levels are associated with aberrant expression of caspase-3 and Creb1 in the early stage of schistosome infection in mice, their potential as diagnostic biomarkers for schistosomiasis hepatopathology progression is unclear [102]. Furthermore, schistosome-specific miRNAs, such as sja-miR-277 and sja-miR-3479-3p, showed potential as biomarkers for diagnosing *S. japonicum* infection and liver fibrosis intensity based on observations in two murine models [104]. Bantam and miR-2c-3p isolated from serum extracellular vesicles of infected patients can also be used as diagnostic and follow-up tools [106]. These findings indicate that circulating miRNAs showed potential as predictors of fibrosis progression, but available information remains limited; at this stage, usage of circulating host miRNAs for schistosomiasis diagnosis is still questionable, because host miRNAs may be altered under a wide range of etiology, thus leading to the problem of diagnostic non-specificity of schistosomiasis. Therefore, more potential miRNAs must be identified that are specific for grading schistosomiasis-associated fibrosis by increasing clinical sample numbers and/or testing extracellular vesicle-derived miRNAs.

## Use of miRNAs to treat schistosomiasis-associated hepatic fibrosis

Regarding treatment of schistosomiasis-associated liver fibrosis with chemical drugs, the function of renin-angiotensin system (RAS) inhibitors and kaempferol in alleviating hepatic fibrosis have been extensively studied. These drugs can inhibit HSC activation and reduce collagen and TGF-β production [107, 108]. Both taurine supplementation and combining PZQ with silymarin substantially ameliorate liver fibrosis, likely by downregulating relevant cytokines or chemokines and reducing the endoplasmic reticular stress response [109, 110]. Mesenchymal stem cell therapy can significantly improve and reverse fibrosis in liver tissues of *S. mansoni*-infected mice [111]. A double-stranded oligodeoxynucleotide decoy containing the TGF-β regulatory element in the distal promoter of the COL1A1 gene was reported to effectively treat schistosome-induced fibrosis by suppressing TGF-β1 and COL1A1 production [112].

MiRNA-based treatments may provide promising prospects for treating schistosomiasis-associated liver fibrosis. Recently, miRNA intervention therapy has been investigated in murine schistosomiasis by delivering miRNA antagonists or mimics. First, vector-based miRNA inhibition, a miRNA silencing strategy, has been tested in mice. Lentivirus or adenovirus vectors are commonly used to deliver miRNA-expression cassettes into target cell lines or animals [113]. In mouse models of schistosomiasis-associated liver fibrosis, inhibiting miR-96 or miR-21 *via* recombinant adeno-associated virus serotype 8 (rAAV8)-mediated delivery of Tough Decoy RNAs can effectively alleviate hepatic fibrosis by reducing collagen I and III [40, 41]. When recombinant lentivirus of let-7b (lenti-let-7b) was transfected into *S. japonicum*-infected mice, the expressions of TGF-β1, TβRI, α-SMA, collagen I, serum IL-4 and IFN-γ were significantly decreased, and liver fibrosis was significantly ameliorated [80]. Furthermore, rAAV8-mediated miR-203-3p elevation could act as a therapeutic intervention for schistosome-induced fibrotic diseases [67]. Secondly, competing endogenous RNAs (ceRNAs) can bind to miRNA through miRNA response elements (MREs), thereby affecting the miRNA-induced gene silencing [114]. Thus, we hypothesize that expression of some key genes involved in schistosomiasis-associated liver fibrosis could be regulated *via* a ceRNA network to alleviate or even cure liver fibrosis. This hypothesis needs to be examined in future studies.

Although miRNAs have shown great potential in treating schistosome-induced hepatic fibrosis, current studies are primarily limited to murine schistosomiasis; therefore, developing therapeutics using anti-miRs or miRNA mimics from bench to clinical trials will take much more time. More importantly, compared with classic drugs, miRNA-based treatment may produce off-target effects, leading to undesired changes in unrelated gene expression [115, 116]. To decrease the unwanted side-effects, delivery of anti-miRs or miRNA mimics to specific cells or tissues is important. As described previously, adeno-associated virus (AAV) remains the primary vector for delivering the miRNA of interest to specific organs. The serotype AAV8 in particular, shows excellent liver specificity owing to its natural tropism towards the liver; therefore, AAV8 was still the major choice in several studies of miRNA-based treatment of liver fibrosis in murine models of schistosomiasis [40, 41, 67]. Furthermore, some new miRNA delivery systems, which exhibited good cell-target efficiency, have been developed [117, 118]. A pH-sensitive and vitamin A (VA)-conjugated copolymer VA-PEG-Bpei-PAsp(DIP-BzA) (abbreviated as T-PBP) was synthesized, and this copolymer was assembled into superparamagnetic iron oxide (SPIO)-decorated cationic micelles, which efficiently transported miRNA-29b and miRNA-122 to HSCs and displayed prominent anti-fibrotic efficacy [117]. A novel lactosylated PDMAEMA nanoparticles efficiently delivered a miR-146b mimic to hepatocytes to alleviate hepatic steatosis in the non-alcoholic fatty liver disease (NAFLD) mouse model [118]. However, the T-PBP micelle and lactosylated PDMAEMA nanoparticles have not been used to deliver miRNAs to treat liver fibrosis in a murine schistosomiasis model.

## Conclusions

Although numerous studies have been conducted to determine the roles of miRNAs in the pathogenesis of schistosomiasis, the current understanding of the miRNA-mediated molecular mechanisms remains limited. Previous studies on liver and serum miRNA expression profiles in murine schistosomiasis have provided valuable information for understanding the pathogenesis of the disease. Some differentially expressed miRNAs exert both pro-fibrogenic and anti-fibrogenic roles during liver pathology progression in schistosomiasis. In order to develop an effective strategy to treat liver fibrosis, miRNA-based intervention has shown great potential to inhibit the progression of chronic schistosomiasis. Although some reports have suggested that intervention of dysregulated hepatic fibrosis-associated specific miRNAs have significant effects in treating schistosomiasis, these studies remain at the animal experimental stage. Safety and effectiveness issues of miRNA therapeutics in schistosomiasis-associated liver fibrosis require further study. Specifically, off-target effects and therapeutic specificity are of considerable concerns. Thus, more biosafety and hepatotropic materials are needed to be developed, and nanoparticles may be good candidates. In addition, circulating miRNAs have become promising biomarkers for grading liver fibrosis in schistosomiasis. In the future, more efforts are needed to clarify the mechanisms of host-parasite interactions and miRNA-mediated liver pathology. Although limited progress has been achieved using intervening single miRNAs to treat schistosomiasis-associated liver fibrosis, great interest exists surrounding schistosomiasis-related miRNAs as a novel therapeutic strategy. It is predictable that more miRNA therapeutic targets will likely be discovered, and innovation in the areas of specific tissue-targeted miRNA delivery will promote specificity of treatment and reduce off-target effects, thereby maximizing the utility of miRNAs in treating schistosomiasis.

## Data Availability

Not applicable.

## Abbreviations

CCL4: carbon tetrachloride
CERNA: competing endogenous RNA
ECM: extracellular matrix
HSC: hepatic stellate cells
IL: interleukin
IFN-γ: interferon-γ
ILC2s: group 2 innate lymphoid cells
IRF2: IFN-regulatory factor 2
Lenti-let-7b: lentivirus of let-7b
LSECs: liver sinusoidal endothelial cells
miRNA: microRNA
MMPs: matrix metallopeptidase
MREs: miRNA response elements
NAFLD: non-alcoholic fatty liver disease
PZQ: Praziquantel
rAAV8: recombinant adeno-associated virus serotype 8
RAS: renin-angiotensin system
SEA: soluble egg antigen
SPIO: superparamagnetic iron oxide
STAT1: activator of transcription 1
TGF-β1: transforming growth factor beta 1
TNF-α: tumor necrosis factor alpha
Tregs: regulatory T cells
UTR: untranslated region
VA: vitamin A
VDR: vitamin D receptor

## References

[1] Egesa M, Hoffmann KF, Hokke CH, Yazdanbakhsh M, Cose S (2017). Rethinking schistosomiasis vaccine development: synthetic vesicles. Trends Parasitol..

[2] Dai Y, Wang X, Tang J, Zhao S, Xing Y, Dai J (2015). Enhancement of protective efficacy through adenoviral vectored vaccine priming and protein boosting strategy encoding triosephosphate isomerase (SjTPI) against *Schistosoma japonicum* in mice. PLoS ONE..

[3] Colley DG, Bustinduy AL, Secor WE, King CH (2014). Human schistosomiasis. Lancet..

[4] Cai P, Liu S, Piao X, Hou N, Gobert GN, McManus DP (2016). Comprehensive transcriptome analysis of sex-biased expressed genes reveals discrete biological and physiological features of male and female *Schistosoma japonicum*. PLoS Negl Trop Dis..

[5] Cai P, Gobert GN, You H, McManus DP (2016). The Tao survivorship of schistosomes: implications for schistosomiasis control. Int J Parasitol..

[6] Pisarski K (2019). The global burden of disease of zoonotic parasitic diseases: top 5 contenders for priority consideration. Trop Med Infect Dis..

[7] da Paz VR, Figueiredo-Vanzan D, dos Santos Pyrrho A (2019). Interaction and involvement of cellular adhesion molecules in the pathogenesis of *Schistosomiasis mansoni*. Immunol Lett..

[8] Cortes-Selva D, Elvington AF, Ready A, Rajwa B, Pearce EJ, Randolph GJ (2018). *Schistosoma mansoni* infection-induced transcriptional changes in hepatic macrophage metabolism correlate with an athero-protective phenotype. Front Immunol..

[9] Zhang B, Wu X, Liu J, Song L, Song Q, Wang L (2019). beta-Actin: not a suitable internal control of hepatic fibrosis caused by *Schistosoma japonicum*. Front Microbiol..

[10] Anthony BJ, Ramm GA, McManus DP (2012). Role of resident liver cells in the pathogenesis of schistosomiasis. Trends Parasitol..

[11] Ni MM, Wang YR, Wu WW, Xia CC, Zhang YH, Xu J (2018). Novel insights on notch signaling pathways in liver fibrosis. Eur J Pharmacol..

[12] He X, Bao J, Chen J, Sun X, Wang J, Zhu D (2015). Adenovirus-mediated over-expression of Septin4 ameliorates hepatic fibrosis in mouse livers infected with *Schistosoma japonicum*. Parasitol Int..

[13] El-Kady AM, Ahmad AA, Hassan TM, El-Deek HEM, Fouad SS, Althagfan SS (2019). Eugenol, a potential schistosomicidal agent with anti-inflammatory and antifibrotic effects against *Schistosoma mansoni*, induced liver pathology. Infect Drug Resist..

[14] Ma Z, Liu X, Dong H, Xia D, Wang L, Chen Y (2018). Sorafenib and praziquantel synergistically attenuate *Schistosoma japonicum*-induced liver fibrosis in mice. Parasitol Res..

[15] Hagen J, Scheerlinck JP, Gasser RB (2015). Knocking down schistosomes - promise for lentiviral transduction in parasites. Trends Parasitol..

[16] Liang YJ, Luo J, Yuan Q, Zheng D, Liu YP, Shi L (2011). New insight into the antifibrotic effects of praziquantel on mice in infection with *Schistosoma japonicum*. PLoS ONE..

[17] Kong D, Zhou C, Guo H, Wang W, Qiu J, Liu X (2017). Praziquantel targets M1 macrophages and ameliorates splenomegaly in chronic schistosomiasis. Antimicrob Agents Chemother..

[18] El-Beshbishi SN, Saleh NE, Abd El-Mageed SA, El-Nemr HEE, Abdalla HA, Shebl AM (2019). Effect of omega-3 fatty acids administered as monotherapy or combined with artemether on experimental *Schistosoma mansoni* infection. Acta Trop..

[19] Gouveia MJ, Brindley PJ, Rinaldi G, Gartner F, da Costa JM, Vale N (2019). Combination anthelmintic/antioxidant activity against *Schistosoma Mansoni*. Biomolecules..

[20] Benna C, Rajendran S, Rastrelli M, Mocellin S (2019). miRNA deregulation targets specific pathways in leiomyosarcoma development: an *in silico* analysis. J Transl Med..

[21] Zhang X, Hua F, Yang Z, Chen Y, Teng X, Huang H (2018). Enhancement of immunoregulatory function of modified bone marrow mesenchymal stem cells by targeting SOCS1. Biomed Res Int..

[22] Liu C, Yang H, Shi W, Wang T, Ruan Q (2018). MicroRNA-mediated regulation of T helper type 17/regulatory T-cell balance in autoimmune disease. Immunology..

[23] Queiroz FR, Silva LM, Jeremias WJ, Baba EH, Caldeira RL, Coelho PMZ (2017). Differential expression of small RNA pathway genes associated with the *Biomphalaria glabrata*/*Schistosoma mansoni* interaction. PLoS ONE..

[24] Rouas R, Merimi M, Najar M, El Zein N, Fayyad-Kazan M, Berehab M (2019). Human CD8(+) CD25 (+) CD127 (low) regulatory T cells: microRNA signature and impact on TGF-beta and IL-10 expression. J Cell Physiol..

[25] Tufekci KU, Oner MG, Meuwissen RL, Genc S (2014). The role of microRNAs in human diseases. Methods Mol Biol..

[26] Van Roosbroeck K, Calin GA (2017). Cancer hallmarks and microRNAs: the therapeutic connection. Adv Cancer Res..

[27] Chen JQ, Papp G, Szodoray P, Zeher M (2016). The role of microRNAs in the pathogenesis of autoimmune diseases. Autoimmun Rev..

[28] Arora N, Tripathi S, Singh AK, Mondal P, Mishra A, Prasad A (2017). Micromanagement of immune system: role of miRNAs in helminthic infections. Front Microbiol..

[29] Hong Y, Fu Z, Cao X, Lin J (2017). Changes in microRNA expression in response to *Schistosoma japonicum* infection. Parasite Immunol..

[30] Cai P, Piao X, Liu S, Hou N, Wang H, Chen Q (2013). MicroRNA-gene expression network in murine liver during *Schistosoma japonicum* infection. PLoS ONE..

[31] Yanguas SC, Cogliati B, Willebrords J, Maes M, Colle I, van den Bossche B (2016). Experimental models of liver fibrosis. Arch Toxicol..

[32] Ying HZ, Chen Q, Zhang WY, Zhang HH, Ma Y, Zhang SZ (2017). PDGF signaling pathway in hepatic fibrosis pathogenesis and therapeutics. Mol Med Rep..

[33] Caviglia JM, Yan J, Jang MK, Gwak GY, Affo S, Yu L (2018). MicroRNA-21 and Dicer are dispensable for hepatic stellate cell activation and the development of liver fibrosis. Hepatology..

[34] Wei J, Feng L, Li Z, Xu G, Fan X (2013). MicroRNA-21 activates hepatic stellate cells *via* PTEN/Akt signaling. Biomed Pharmacother..

[35] Afonso MB, Rodrigues PM, Simao AL, Gaspar MM, Carvalho T, Borralho P (2018). miRNA-21 ablation protects against liver injury and necroptosis in cholestasis. Cell Death Differ..

[36] Chen RX, Xia YH, Xue TC, Ye SL (2012). Suppression of microRNA-96 expression inhibits the invasion of hepatocellular carcinoma cells. Mol Med Rep..

[37] Li Z, Wang Y (2018). miR-96 targets SOX6 and promotes proliferation, migration, and invasion of hepatocellular carcinoma. Biochem Cell Biol..

[38] Chen BL, Peng J, Li QF, Yang M, Wang Y, Chen W (2013). Exogenous bone morphogenetic protein-7 reduces hepatic fibrosis in *Schistosoma japonicum*-infected mice *via* transforming growth factor-beta/Smad signaling. World J Gastroenterol..

[39] Carson JP, Ramm GA, Robinson MW, McManus DP, Gobert GN (2018). Schistosome-induced fibrotic disease: the role of hepatic stellate cells. Trends Parasitol..

[40] He X, Xie J, Zhang D, Su Q, Sai X, Bai R (2015). Recombinant adeno-associated virus-mediated inhibition of microRNA-21 protects mice against the lethal schistosome infection by repressing both IL-13 and transforming growth factor beta 1 pathways. Hepatology..

[41] Luo X, Zhang D, Xie J, Su Q, He X, Bai R (2018). MicroRNA-96 promotes schistosomiasis hepatic fibrosis in mice by suppressing Smad7. Mol Ther Methods Clin Dev..

[42] He Q, Qiu J, Dai M, Fang Q, Sun X, Gong Y (2016). MicroRNA-351 inhibits denervation-induced muscle atrophy by targeting TRAF6. Exp Ther Med..

[43] Li X, Feng R, Huang C, Wang H, Wang J, Zhang Z (2012). MicroRNA-351 regulates TMEM 59 (DCF1) expression and mediates neural stem cell morphogenesis. RNA Biol..

[44] Zhang LN, Tian H, Zhou XL, Tian SC, Zhang XH, Wu TJ (2018). Upregulation of microRNA-351 exerts protective effects during sepsis by ameliorating skeletal muscle wasting through the Tead-4-mediated blockade of the Hippo signaling pathway. FASEB J..

[45] He X, Sun Y, Lei N, Fan X, Zhang C, Wang Y (2018). MicroRNA-351 promotes schistosomiasis-induced hepatic fibrosis by targeting the vitamin D receptor. Proc Natl Acad Sci USA.

[46] He X, Tang R, Sun Y, Wang YG, Zhen KY, Zhang DM (2016). MicroR-146 blocks the activation of M1 macrophage by targeting signal transducer and activator of transcription 1 in hepatic schistosomiasis. EBioMedicine..

[47] Heymann F, Trautwein C, Tacke F (2009). Monocytes and macrophages as cellular targets in liver fibrosis. Inflamm Allergy Drug Targets..

[48] Zhang CY, Yuan WG, He P, Lei JH, Wang CX (2016). Liver fibrosis and hepatic stellate cells: etiology, pathological hallmarks and therapeutic targets. World J Gastroenterol..

[49] Herbert DR, Holscher C, Mohrs M, Arendse B, Schwegmann A, Radwanska M (2004). Alternative macrophage activation is essential for survival during schistosomiasis and downmodulates T helper 1 responses and immunopathology. Immunity..

[50] Reyes JL, Terrazas LI (2007). The divergent roles of alternatively activated macrophages in helminthic infections. Parasite Immunol..

[51] Ricardo SD, van Goor H, Eddy AA (2008). Macrophage diversity in renal injury and repair. J Clin Invest..

[52] Pesce JT, Ramalingam TR, Mentink-Kane MM, Wilson MS, El Kasmi KC, Smith AM (2009). Arginase-1-expressing macrophages suppress Th2 cytokine-driven inflammation and fibrosis. PLoS Pathog..

[53] Dias MV, Castro AP, Campos CC, Souza-Silva TG, Goncalves RV, Souza RLM (2019). Doxycycline hyclate: A schistosomicidal agent *in vitro* with immunomodulatory potential on granulomatous inflammation *in vivo*. Int Immunopharmacol..

[54] Barron L, Wynn TA (2011). Macrophage activation governs schistosomiasis-induced inflammation and fibrosis. Eur J Immunol..

[55] Zhu Y, Ni Y, Liu R, Hou M, Yang B, Song J (2018). PPAR-gamma agonist alleviates liver and spleen pathology *via* inducing Treg cells during *Schistosoma japonicum* infection. J Immunol Res..

[56] Gong W, Huang F, Sun L, Yu A, Zhang X, Xu Y (2018). Toll-like receptor-2 regulates macrophage polarization induced by excretory-secretory antigens from *Schistosoma japonicum* eggs and promotes liver pathology in murine schistosomiasis. PLoS Negl Trop Dis..

[57] Liu L, Jin M, Tao Q, Yu L, Du J, Wang C (2018). Effective amelioration of liver fibrosis through lentiviral vector carrying *Toxoplasma gondii* gra15II in murine model. Front Immunol..

[58] Everts B (2016). Micro(RNAs)managing macrophage polarization during schistosomiasis. EBioMedicine..

[59] Yang Z, Peng Y, Yang S (2019). MicroRNA-146a regulates the transformation from liver fibrosis to cirrhosis in patients with hepatitis B *via* interleukin-6. Exp Ther Med..

[60] Zou Y, Cai Y, Lu D, Zhou Y, Yao Q, Zhang S (2017). MicroRNA-146a-5p attenuates liver fibrosis by suppressing profibrogenic effects of TGFbeta1 and lipopolysaccharide. Cell Signal..

[61] Self-Fordham JB, Naqvi AR, Uttamani JR, Kulkarni V, Nares S (2017). MicroRNA: Dynamic regulators of Macrophage polarization and plasticity. Front Immunol..

[62] Gonzalez-Polo V, Pucci-Molineris M, Cervera V, Gambaro S, Yantorno SE, Descalzi V (2019). Group 2 innate lymphoid cells exhibit progressively higher levels of activation during worsening of liver fibrosis. Ann Hepatol..

[63] Kotsiou OS, Gourgoulianis KI, Zarogiannis SG (2018). IL-33/ST2 axis in organ fibrosis. Front Immunol..

[64] Shieh JM, Tseng HY, Jung F, Yang SH, Lin JC (2019). Elevation of IL-6 and IL-33 levels in serum associated with lung fibrosis and skeletal muscle wasting in a bleomycin-induced lung injury mouse model. Mediators Inflamm..

[65] Liu L, Mao L, Wu X, Wu T, Liu W, Yang Y (2019). BRG1 regulates endothelial-derived IL-33 to promote ischemia-reperfusion induced renal injury and fibrosis in mice. Biochim Biophys Acta Mol Basis Dis..

[66] Garbern JC, Williams J, Kristl AC, Malick A, Rachmin I, Gaeta B (2019). Dysregulation of IL-33/ST2 signaling and myocardial periarteriolar fibrosis. J Mol Cell Cardiol..

[67] He X, Xie J, Wang Y, Fan X, Su Q, Sun Y (2018). Down-regulation of microRNA-203-3p initiates type 2 pathology during schistosome infection *via* elevation of interleukin-33. PLoS Pathog..

[68] Li ZY, Xiao L, Lin G, Tang J, Chen Y, Chen L (2019). Contribution of tissue transglutaminase to the severity of hepatic fibrosis resulting from *Schistosoma japonicum* infection through the regulation of IL-33/ST2 expression. Parasit Vectors..

[69] Ding N, Wang Y, Dou C, Liu F, Guan G, Wei K (2019). Physalin D regulates macrophage M1/M2 polarization *via* the STAT1/6 pathway. J Cell Physiol..

[70] Hu D, Hu Y, Xu W, Yu H, Yang N, Ni S (2017). miR203 inhibits the expression of collagenrelated genes and the proliferation of hepatic stellate cells through a SMAD3dependent mechanism. Mol Med Rep..

[71] Yang X, Li X, Lin Q, Xu Q (2019). Up-regulation of microRNA-203 inhibits myocardial fibrosis and oxidative stress in mice with diabetic cardiomyopathy through the inhibition of PI3K/Akt signaling pathway *via* PIK3CA. Gene..

[72] Wu Y, Zhong JL, Hou N, Sun Y, Ma B, Nisar MF (2017). MicroRNA Let-7b inhibits keratinocyte migration in cutaneous wound healing by targeting IGF2BP2. Exp Dermatol..

[73] Wu Y, Liu L, Bian C, Diao Q, Nisar MF, Jiang X (2018). MicroRNA let-7b inhibits keratinocyte differentiation by targeting IL-6 mediated ERK signaling in psoriasis. Cell Commun Signal..

[74] Wang M, Ouyang J, Li H (2019). CERNA2: A predictor for clinical progression and poor prognosis in cervical carcinoma. J Cell Biochem..

[75] Li H, Zhao L, Zhang Z, Zhang H, Ding C, Su Z (2017). Roles of microRNA let-7b in papillary thyroid carcinoma by regulating HMGA2. Tumour Biol..

[76] Lu PW, Li L, Wang F, Gu YT (2018). Effects of long non-coding RNA HOST2 on cell migration and invasion by regulating MicroRNA let-7b in breast cancer. J Cell Biochem..

[77] Nishi M, Eguchi-Ishimae M, Wu Z, Gao W, Iwabuki H, Kawakami S (2013). Suppression of the let-7b microRNA pathway by DNA hypermethylation in infant acute lymphoblastic leukemia with MLL gene rearrangements. Leukemia..

[78] Hou W, Tian Q, Steuerwald NM, Schrum LW, Bonkovsky HL (2012). The let-7 microRNA enhances heme oxygenase-1 by suppressing Bach1 and attenuates oxidant injury in human hepatocytes. Biochim Biophys Acta..

[79] McDaniel K, Huang L, Sato K, Wu N, Annable T, Zhou T (2017). The let-7/Lin28 axis regulates activation of hepatic stellate cells in alcoholic liver injury. J Biol Chem..

[80] Tang N, Wu Y, Cao W, Liang Y, Gao Y, Hu L (2017). Lentivirus-mediated over-expression of let-7b microRNA suppresses hepatic fibrosis in the mouse infected with *Schistosoma japonicum*. Exp Parasitol..

[81] Zhou J, Li X, Wu X, Zhang T, Zhu Q, Wang X (2018). Exosomes released from tumor-associated macrophages transfer miRNAs that induce a Treg/Th17 cell imbalance in epithelial ovarian cancer. Cancer Immunol Res..

[82] Pei X, Wang X, Li H (2018). LncRNA SNHG1 regulates the differentiation of Treg cells and affects the immune escape of breast cancer *via* regulating miR-448/IDO. Int J Biol Macromol..

[83] Finlay CM, Walsh KP, Mills KH (2014). Induction of regulatory cells by helminth parasites: exploitation for the treatment of inflammatory diseases. Immunol Rev..

[84] Zhou S, Qi Q, Wang X, Zhang L, Xu L, Dong L (2018). SjHSP60 induces CD4(+) CD25(+) Foxp3(+) Tregs *via* TLR4-Mal-drived production of TGF-beta in macrophages. Immunol Cell Biol..

[85] Chuah C, Jones MK, Burke ML, McManus DP, Gobert GN (2014). Cellular and chemokine-mediated regulation in schistosome-induced hepatic pathology. Trends Parasitol..

[86] Singh KP, Gerard HC, Hudson AP, Reddy TR, Boros DL (2005). Retroviral Foxp3 gene transfer ameliorates liver granuloma pathology in *Schistosoma mansoni* infected mice. Immunology..

[87] Anandagoda N, Willis JC, Hertweck A, Roberts LB, Jackson I, Gokmen MR (2019). microRNA-142-mediated repression of phosphodiesterase 3B critically regulates peripheral immune tolerance. J Clin Invest..

[88] Kelada S, Sethupathy P, Okoye IS, Kistasis E, Czieso S, White SD (2013). miR-182 and miR-10a are key regulators of Treg specialisation and stability during schistosome and leishmania-associated inflammation. PLoS Pathog..

[89] Zhu D, Lyu L, Shen P, Wang J, Chen J, Sun X (2018). rSjP40 protein promotes PPARgamma expression in LX-2 cells through microRNA-27b. FASEB J..

[90] Guo CJ, Pan Q, Li DG, Sun H, Liu BW (2009). miR-15b and miR-16 are implicated in activation of the rat hepatic stellate cell: an essential role for apoptosis. J Hepatol..

[91] Zhu D, He X, Duan Y, Chen J, Wang J, Sun X (2014). Expression of microRNA-454 in TGF-beta1-stimulated hepatic stellate cells and in mouse livers infected with *Schistosoma japonicum*. Parasit Vectors..

[92] Dai W, Zhao J, Tang N, Zeng X, Wu K, Ye C (2015). MicroRNA-155 attenuates activation of hepatic stellate cell by simultaneously preventing EMT process and ERK1 signalling pathway. Liver Int..

[93] Zhu D, Yang C, Shen P, Chen L, Chen J, Sun X (2018). rSjP40 suppresses hepatic stellate cell activation by promoting microRNA-155 expression and inhibiting STAT5 and FOXO3a expression. J Cell Mol Med..

[94] Tao R, Fan XX, Yu HJ, Ai G, Zhang HY, Kong HY (2018). MicroRNA-29b-3p prevents *Schistosoma japonicum*-induced liver fibrosis by targeting COL1A1 and COL3A1. J Cell Biochem..

[95] Zhao Y, Dang Z, Chong S (2019). Mmu-miR-92a-2-5p targets TLR2 to relieve *Schistosoma japonicum*-induced liver fibrosis. Int Immunopharmacol..

[96] Wang M, Abais JM, Meng N, Zhang Y, Ritter JK, Li P-L (2014). Upregulation of cannabinoid receptor-1 and fibrotic activation of mouse hepatic stellate cells during *Schistosoma* J. infection: role of NADPH oxidase. Free Radic Biol Med..

[97] Movahedpour A, Ahmadi N, Ghasemi Y, Savardashtaki A, Shabaninejad Z (2019). Circulating microRNAs as potential diagnostic biomarkers and therapeutic targets in prostate cancer: current status and future perspectives. J Cell Biochem..

[98] Abdollahi A, Rahmati S, Ghaderi B, Sigari N, Nikkhoo B, Sharifi K (2019). A combined panel of circulating microRNA as a diagnostic tool for detection of the non-small cell lung cancer. QJM..

[99] Parizadeh SM, Jafarzadeh-Esfehani R, Ghandehari M, Hasanzadeh M, Parizadeh SMR, Hassanian SM (2019). Circulating and tissue microRNAs as biomarkers for ovarian cancer prognosis. Curr Drug Targets..

[100] Xu J, Wu C, Che X, Wang L, Yu D, Zhang T (2011). Circulating microRNAs, miR-21, miR-122, and miR-223, in patients with hepatocellular carcinoma or chronic hepatitis. Mol Carcinog..

[101] Wang K, Zhang S, Marzolf B, Troisch P, Brightman A, Hu Z (2009). Circulating microRNAs, potential biomarkers for drug-induced liver injury. Proc Natl Acad Sci USA.

[102] Zhu L, Dao J, Du X, Li H, Lu K, Liu J (2015). Altered levels of circulating miRNAs are associated *Schistosoma japonicum* infection in mice. Parasit Vectors..

[103] He X, Sai X, Chen C, Zhang Y, Xu X, Zhang D (2013). Host serum miR-223 is a potential new biomarker for *Schistosoma japonicum* infection and the response to chemotherapy. Parasit Vectors..

[104] Cai P, Gobert GN, You H, Duke M, McManus DP (2015). Circulating miRNAs: potential novel biomarkers for hepatopathology progression and diagnosis of *Schistosomiasis japonica* in two murine models. PLoS Negl Trop Dis..

[105] Cai P, Mu Y, Olveda RM, Ross AG, Olveda DU, McManus DP (2018). Circulating miRNAs as footprints for liver fibrosis grading in schistosomiasis. Ebiomedicine..

[106] Meningher T, Lerman G, Regev-Rudzki N, Gold D, Ben-Dov IZ, Sidi Y (2017). Schistosomal microRNAs isolated from extracellular vesicles in sera of infected patients: a new tool for diagnosis and follow-up of human schistosomiasis. J Infect Dis..

[107] Zhou YP, Zhang SL, Cheng D, Li HR, Tang ZM, Xue J (2013). Preliminary exploration on anti-fibrosis effect of kaempferol in mice with *Schistosoma japonicum* infection. Eur J Inflamm..

[108] Parreira NA, Ramalho FS, Augusto MJ, Silva DM, Prado CM, Elias Junior J (2018). The comparative efficacy of renin-angiotensin system blockers in schistosomal hepatic fibrosis. Exp Parasitol..

[109] Yu YR, Ni XQ, Huang J, Zhu YH, Qi YF (2016). Taurine drinking ameliorates hepatic granuloma and fibrosis in mice infected with *Schistosoma japonicum*. Int J Parasitol Drugs Drug Resist..

[110] El-Sayed NM, Fathy GM, Abdel-Rahman SA, El-Shafei MA (2016). Cytokine patterns in experimental *Schistosomiasis mansoni* infected mice treated with silymarin. J Parasit Dis..

[111] Hegab MH, Abd-Allah SH, Badawey MS, Saleh AA, Metwally AS, Fathy GM (2018). Therapeutic potential effect of bone marrow-derived mesenchymal stem cells on chronic liver disease in murine *Schistosomiasis mansoni*. J Parasit Dis..

[112] Boros DL, Singh KP, Gerard HC, Hudson AP, White SL, Cutroneo KR (2005). A novel nonsteroidal antifibrotic oligo decoy containing the TGF-beta element found in the COL1A1 gene which regulates murine schistosomiasis liver fibrosis. J Cell Physiol..

[113] Cheng G, Jin Y (2012). MicroRNAs: potentially important regulators for schistosome development and therapeutic targets against schistosomiasis. Parasitology..

[114] Salmena L, Poliseno L, Tay Y, Kats L, Pandolfi PP (2011). A ceRNA hypothesis: the Rosetta Stone of a hidden RNA language. Cell.

[115] van Rooij E, Purcell AL, Levin AA (2012). Developing microRNA therapeutics. Circ Res..

[116] Cai P, Gobert GN, McManus DP (2016). MicroRNAs in parasitic helminthiases: current status and future perspectives. Trends Parasitol..

[117] Wu J, Huang J, Kuang S, Chen J, Li X, Chen B (2019). Synergistic microRNA therapy in liver fibrotic rat using MRI-visible nanocarrier targeting hepatic stellate cells. Adv Sci..

[118] He S, Guo W, Deng F, Chen K, Jiang Y, Dong M (2018). Targeted delivery of microRNA 146b mimic to hepatocytes by lactosylated PDMAEMA nanoparticles for the treatment of NAFLD. Artif Cells Nanomed Biotechnol..

[119] Tang H, Liang YB, Chen ZB, Du LL, Zeng LJ, Wu JG (2017). Soluble egg antigen activates M2 macrophages *via* the STAT6 and PI3K pathways, and *Schistosoma japonicum* alternatively activates macrophage polarization to improve the survival rate of septic mice. J Cell Biochem..

[120] Chu DY, Li CL, Li J, Luo F, Zheng MJ, Wu Q (2008). Effect of paeoniflorin on secretion of TGF-beta1 from macrophages in mice. Zhongguo Ji Sheng Chong Xue Yu Ji Sheng Chong Bing Za Zhi..

